# Supplementary material for: Novel role of bone morphogenetic protein 9 in innate host responses to HCMV infection
Source: EMBO Rep. 2024 Mar 11;25(3):1106–29. doi: 10.1038/s44319-024-00072-2 (PMC10933439; doi:10.1038/s44319-024-00072-2)
Supplement: Supplementary file 1 — Source Data Fig. 2 [file 44319_2024_72_MOESM1_ESM.zip › 2C.pptx]

## Slide 1
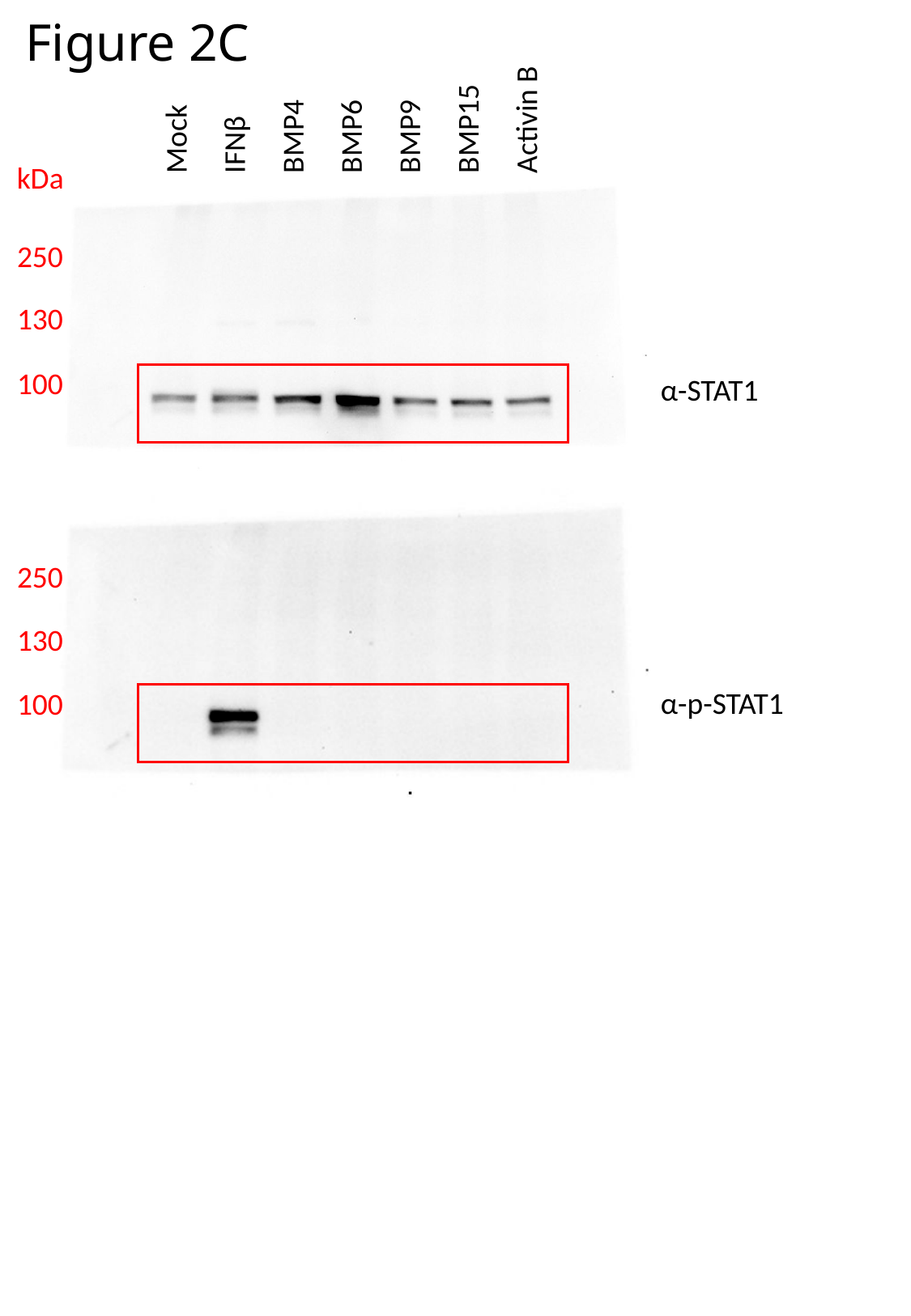

# Figure 2C
Mock
IFNβ
BMP4
BMP6
BMP9
BMP15
Activin B
kDa
250
130
100
α-STAT1
250
130
α-p-STAT1
100

## Slide 2
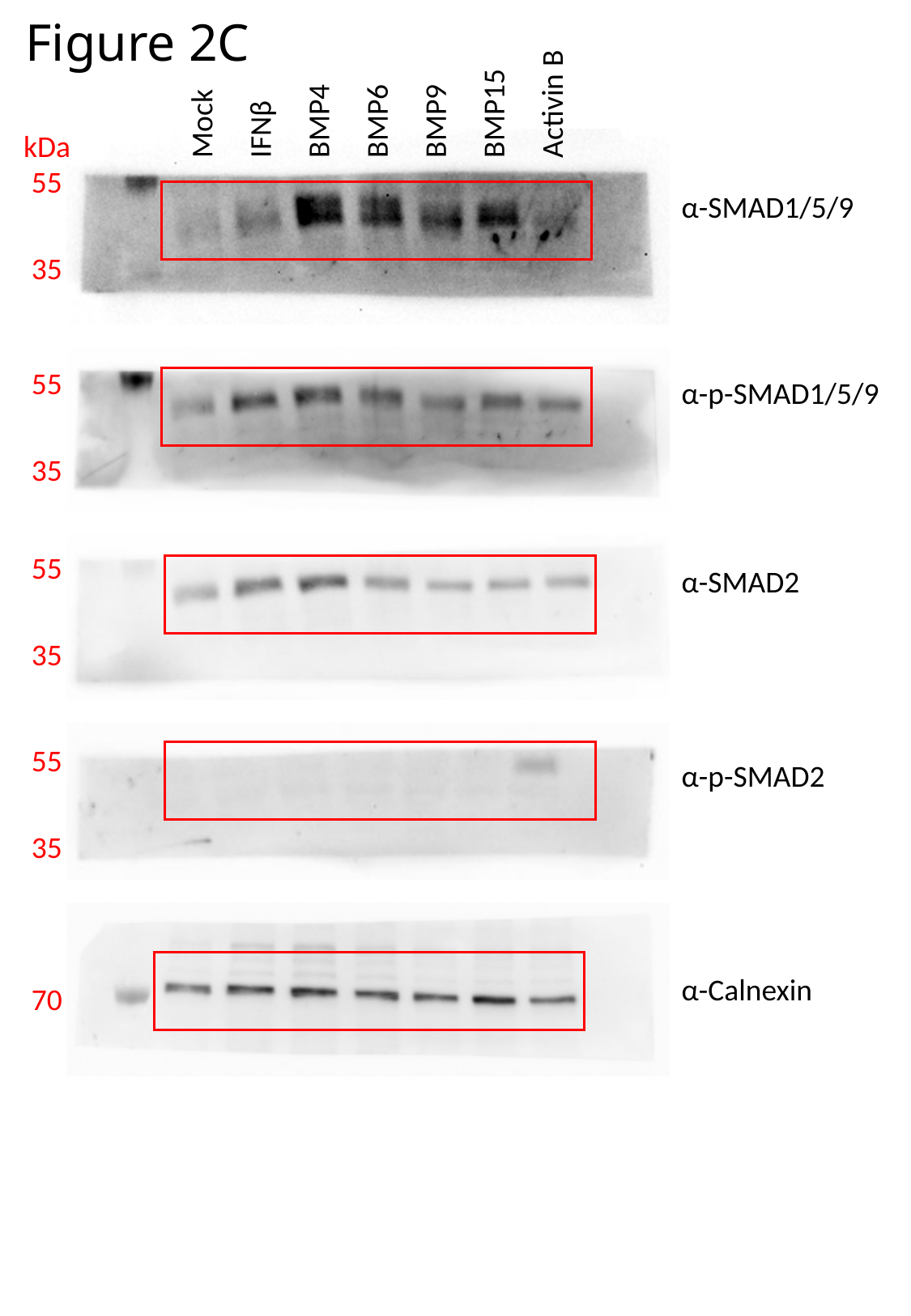

# Figure 2C
Mock
IFNβ
BMP4
BMP6
BMP9
BMP15
Activin B
kDa
55
α-SMAD1/5/9
35
55
α-p-SMAD1/5/9
35
55
α-SMAD2
35
55
α-p-SMAD2
35
α-Calnexin
70
