# Supplementary material for: Novel role of bone morphogenetic protein 9 in innate host responses to HCMV infection
Source: EMBO Rep. 2024 Mar 11;25(3):1106–29. doi: 10.1038/s44319-024-00072-2 (PMC10933439; doi:10.1038/s44319-024-00072-2)
Supplement: Supplementary file 4 — Source Data Fig. 5 [file 44319_2024_72_MOESM4_ESM.zip › 5B_image data.pptx]

## Slide 1
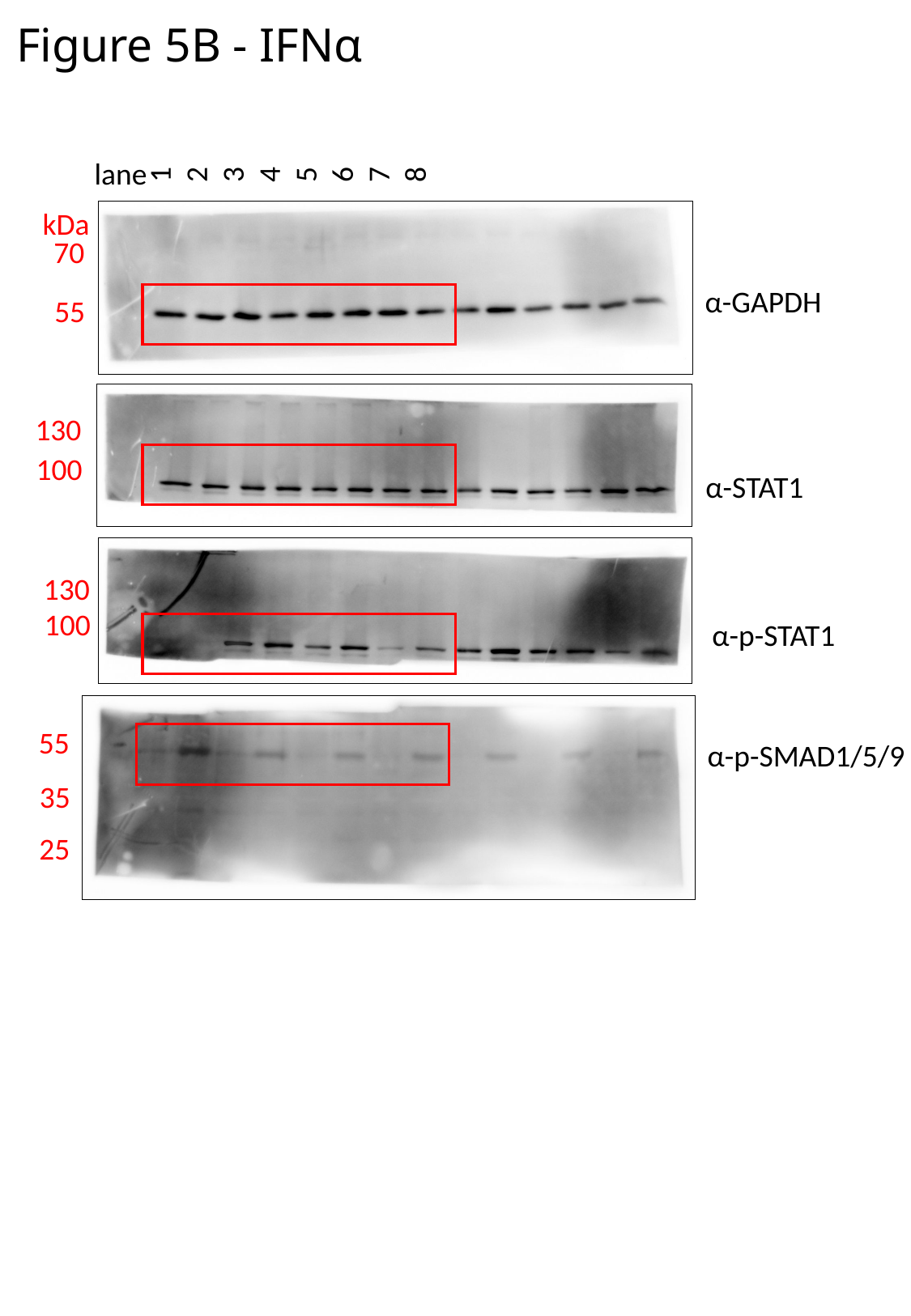

# Figure 5B - IFNα
lane
1
2
3
4
5
6
7
8
kDa
70
α-GAPDH
55
130
100
α-STAT1
130
100
α-p-STAT1
55
α-p-SMAD1/5/9
35
25

## Slide 2
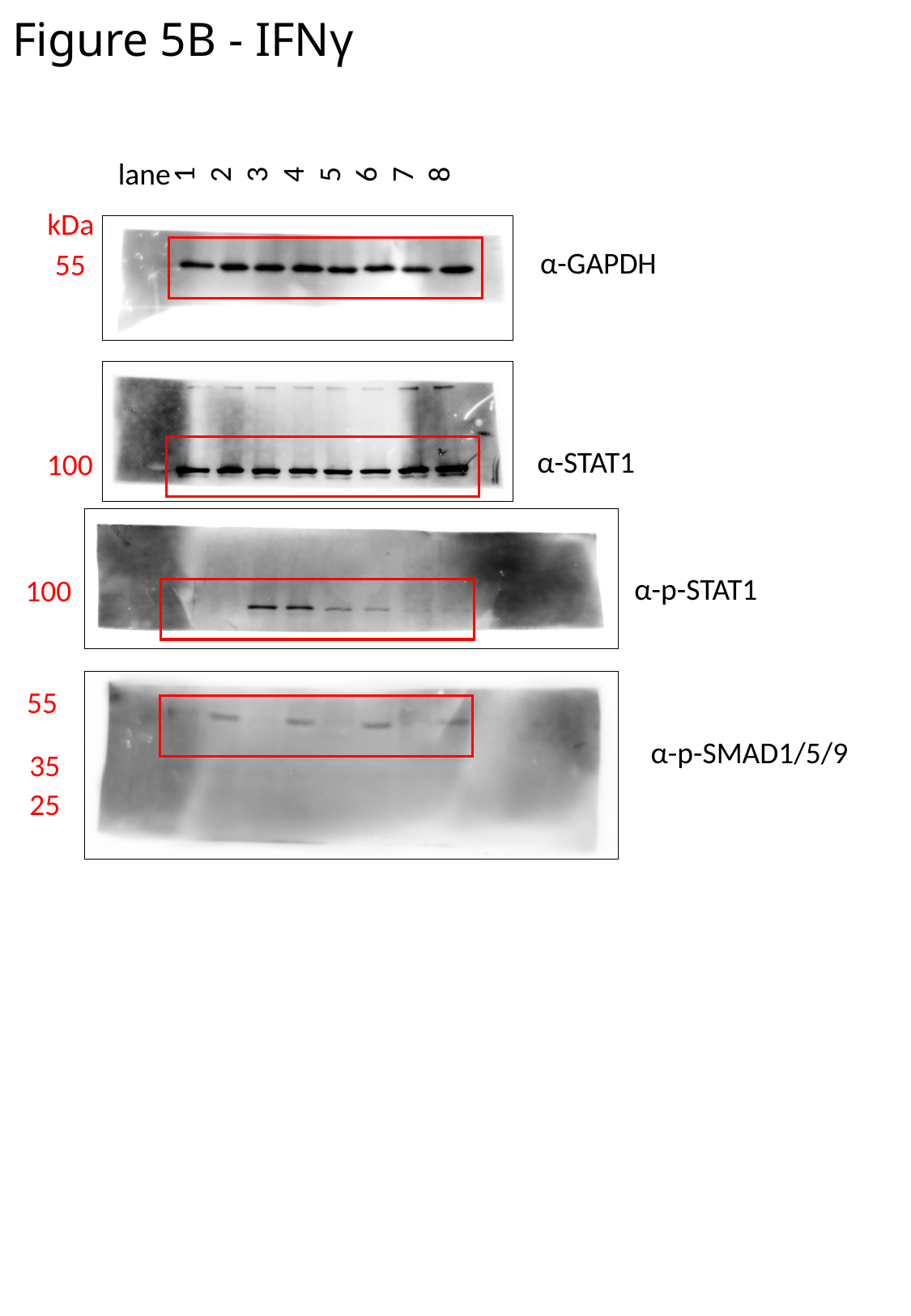

Figure 5B - IFNγ
lane
1
2
3
4
5
6
7
8
kDa
α-GAPDH
55
α-STAT1
100
α-p-STAT1
100
55
α-p-SMAD1/5/9
35
25
