# Supplementary figures and images for: Novel role of bone morphogenetic protein 9 in innate host responses to HCMV infection
Source: EMBO Rep. 2024 Mar 11;25(3):1106–29. doi: 10.1038/s44319-024-00072-2 (PMC10933439; doi:10.1038/s44319-024-00072-2)

## Slide 1
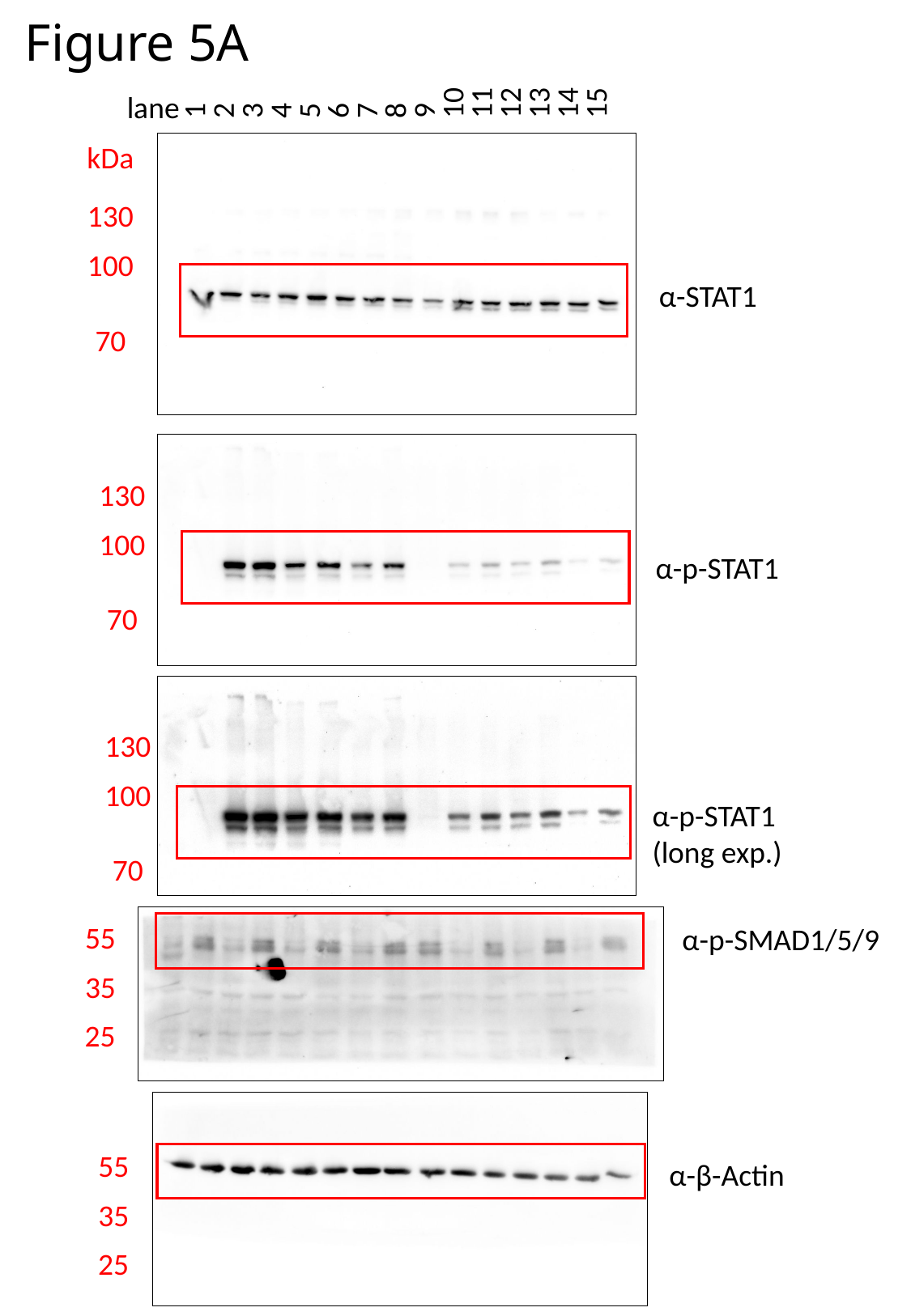

# Figure 5A
lane
1
2
3
4
5
6
7
8
9
10
11
12
13
14
15
kDa
130
100
α-STAT1
70
130
100
α-p-STAT1
70
130
100
α-p-STAT1
(long exp.)
70
55
α-p-SMAD1/5/9
35
25
55
α-β-Actin
35
25

Supplement: Supplementary file 4 — Source Data Fig. 5 [file 44319_2024_72_MOESM4_ESM.zip › 5A_image data.pptx]

## Slide 1
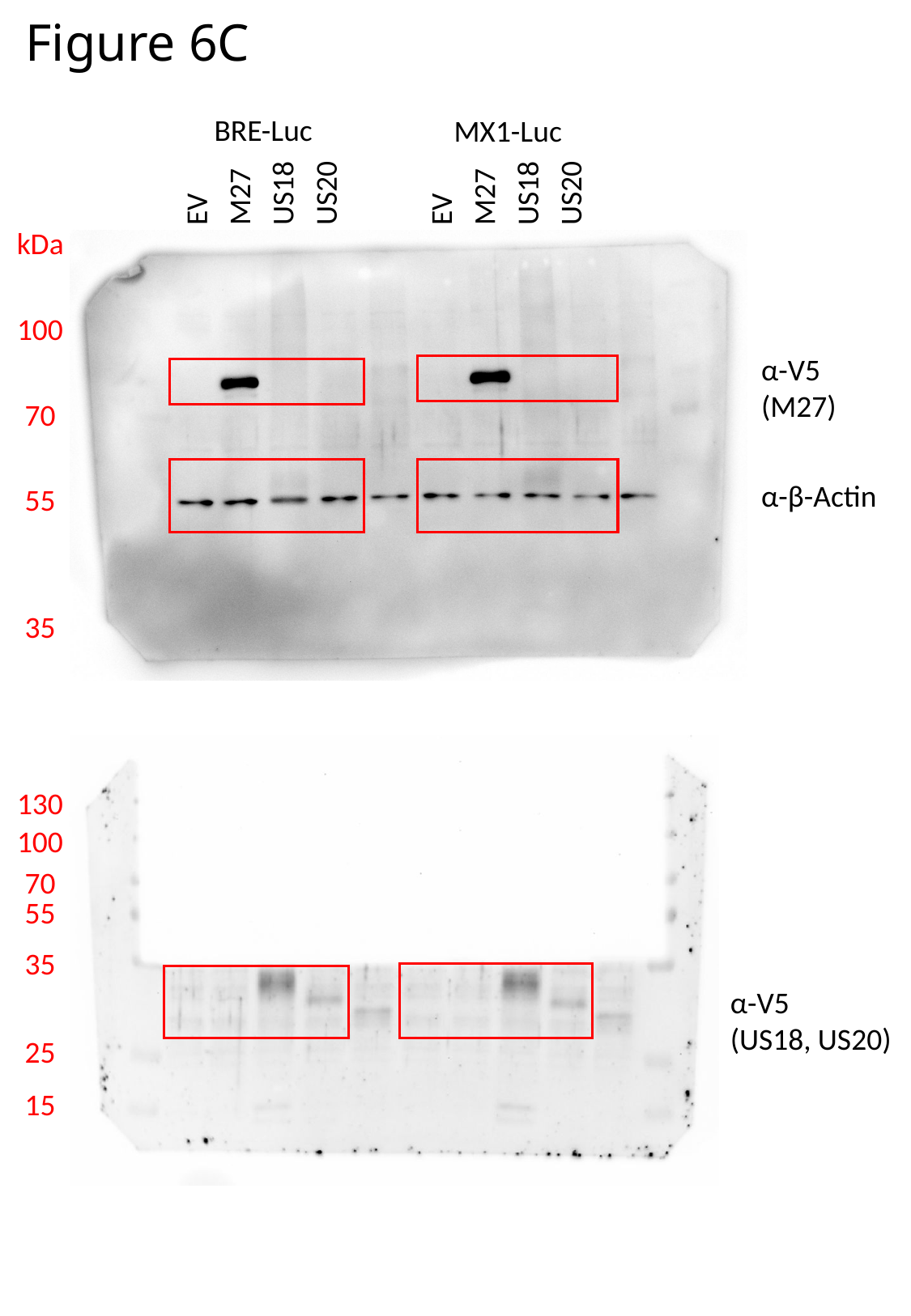

# Figure 6C
BRE-Luc
MX1-Luc
EV
M27
US18
US20
EV
M27
US18
US20
kDa
100
α-V5 (M27)
70
α-β-Actin
55
35
130
100
70
55
35
α-V5
(US18, US20)
25
15

Supplement: Supplementary file 5 — Source Data Fig. 6 [file 44319_2024_72_MOESM5_ESM.zip › 6C.pptx]

## Slide 1
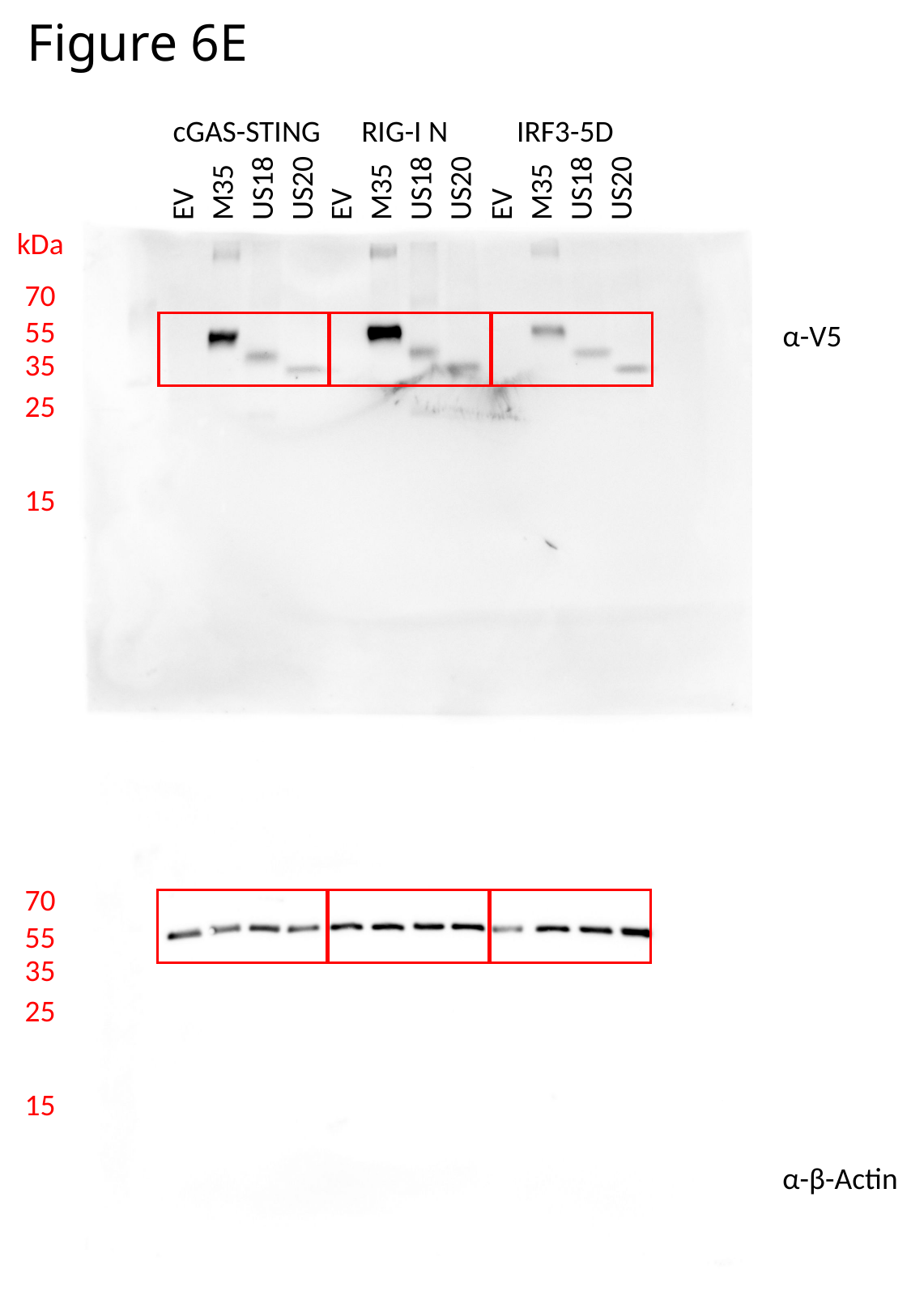

# Figure 6E
cGAS-STING
RIG-I N
IRF3-5D
EV
M35
US18
US20
EV
M35
US18
US20
EV
M35
US18
US20
kDa
70
55
α-V5
35
25
15
70
55
35
25
15
α-β-Actin

Supplement: Supplementary file 5 — Source Data Fig. 6 [file 44319_2024_72_MOESM5_ESM.zip › 6E_image Data.pptx]

## Slide 1
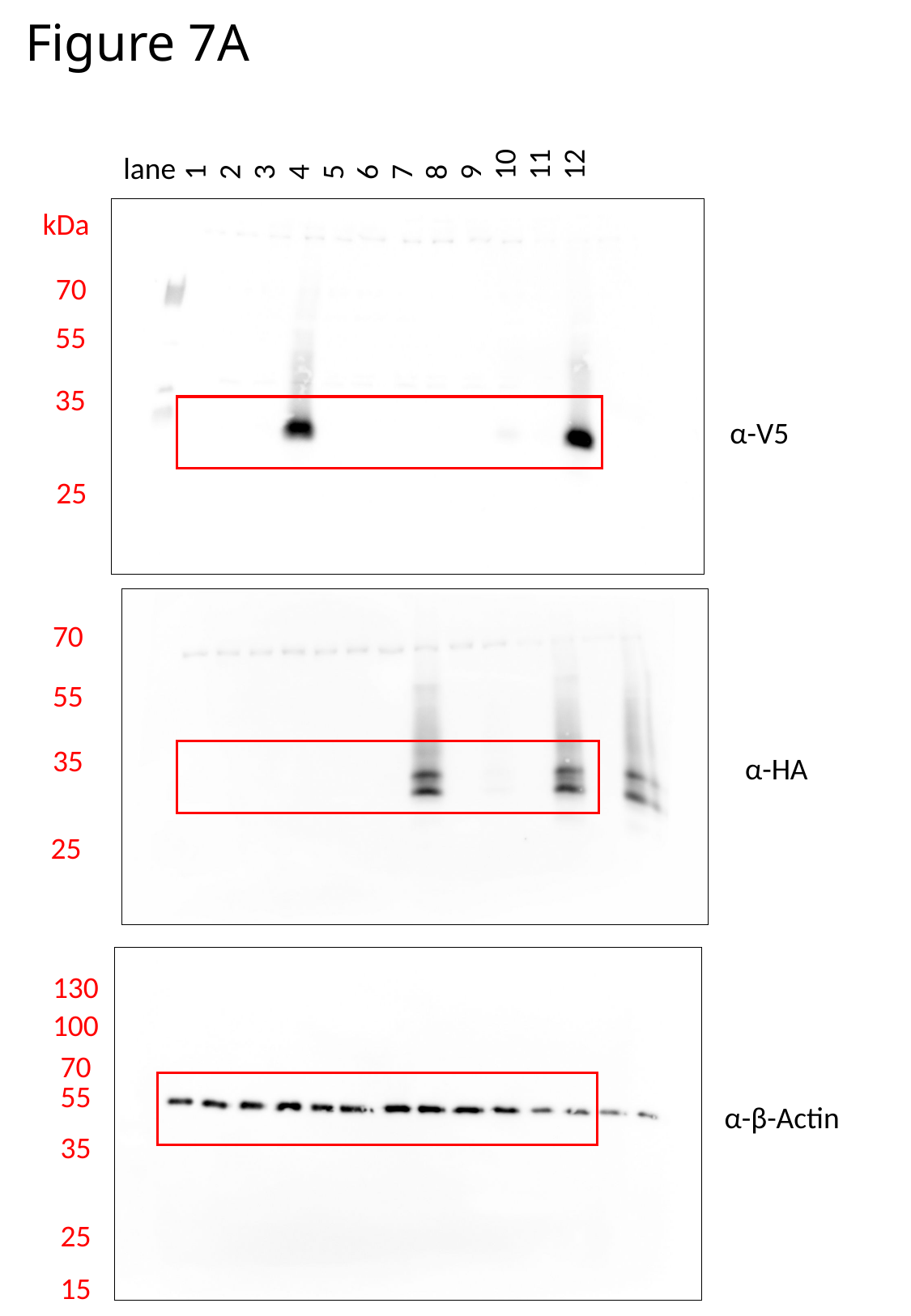

# Figure 7A
lane
1
2
3
4
5
6
7
8
9
10
11
12
kDa
70
55
35
α-V5
25
70
55
35
α-HA
25
130
100
70
55
α-β-Actin
35
25
15

Supplement: Supplementary file 6 — Source Data Fig. 7 [file 44319_2024_72_MOESM6_ESM.zip › 7A.pptx]

## Slide 1
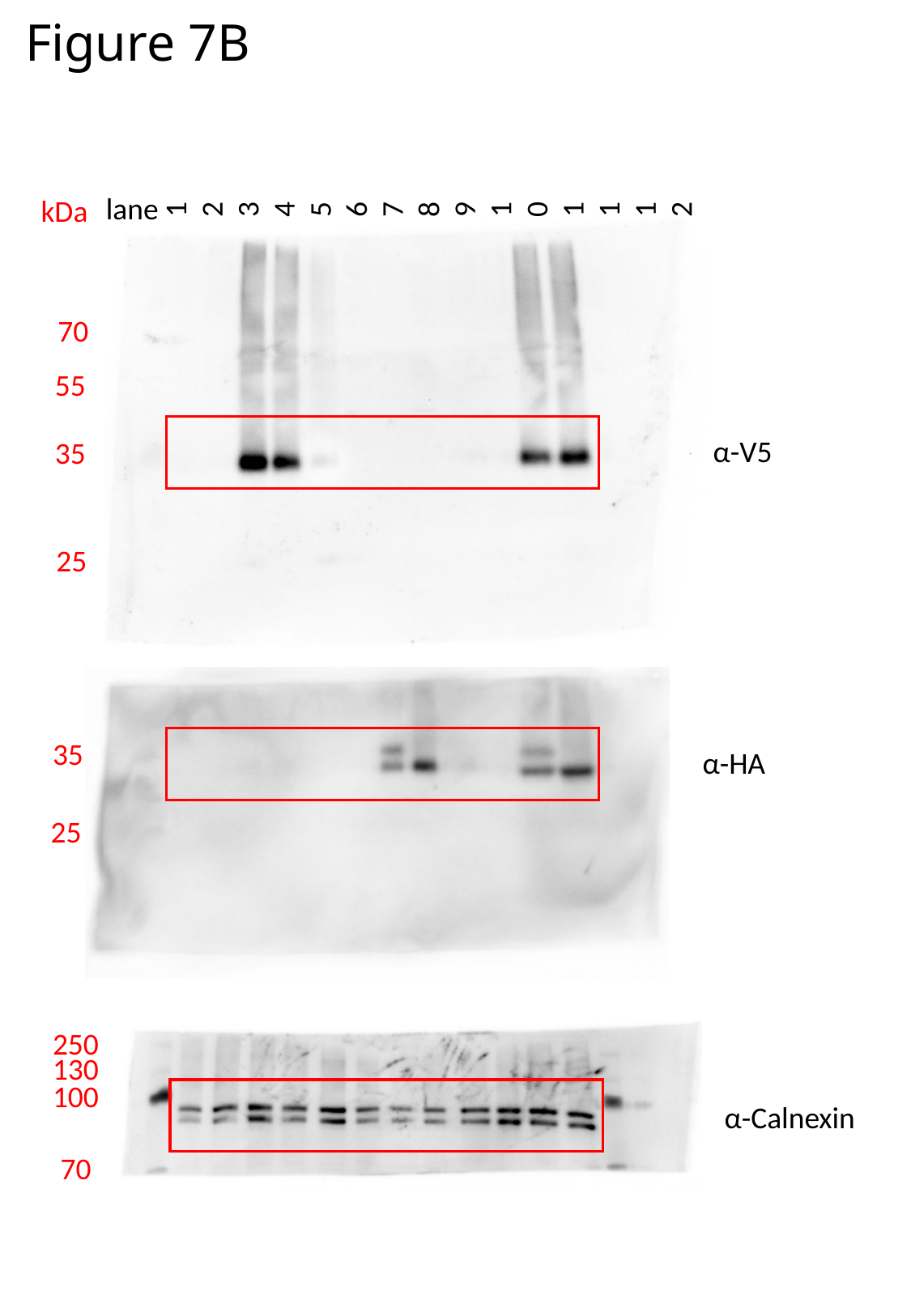

# Figure 7B
lane
1
2
3
4
5
6
7
8
9
10
11
12
kDa
70
55
α-V5
35
25
35
α-HA
25
250
130
100
α-Calnexin
70

Supplement: Supplementary file 6 — Source Data Fig. 7 [file 44319_2024_72_MOESM6_ESM.zip › 7B.pptx]

## Slide 1
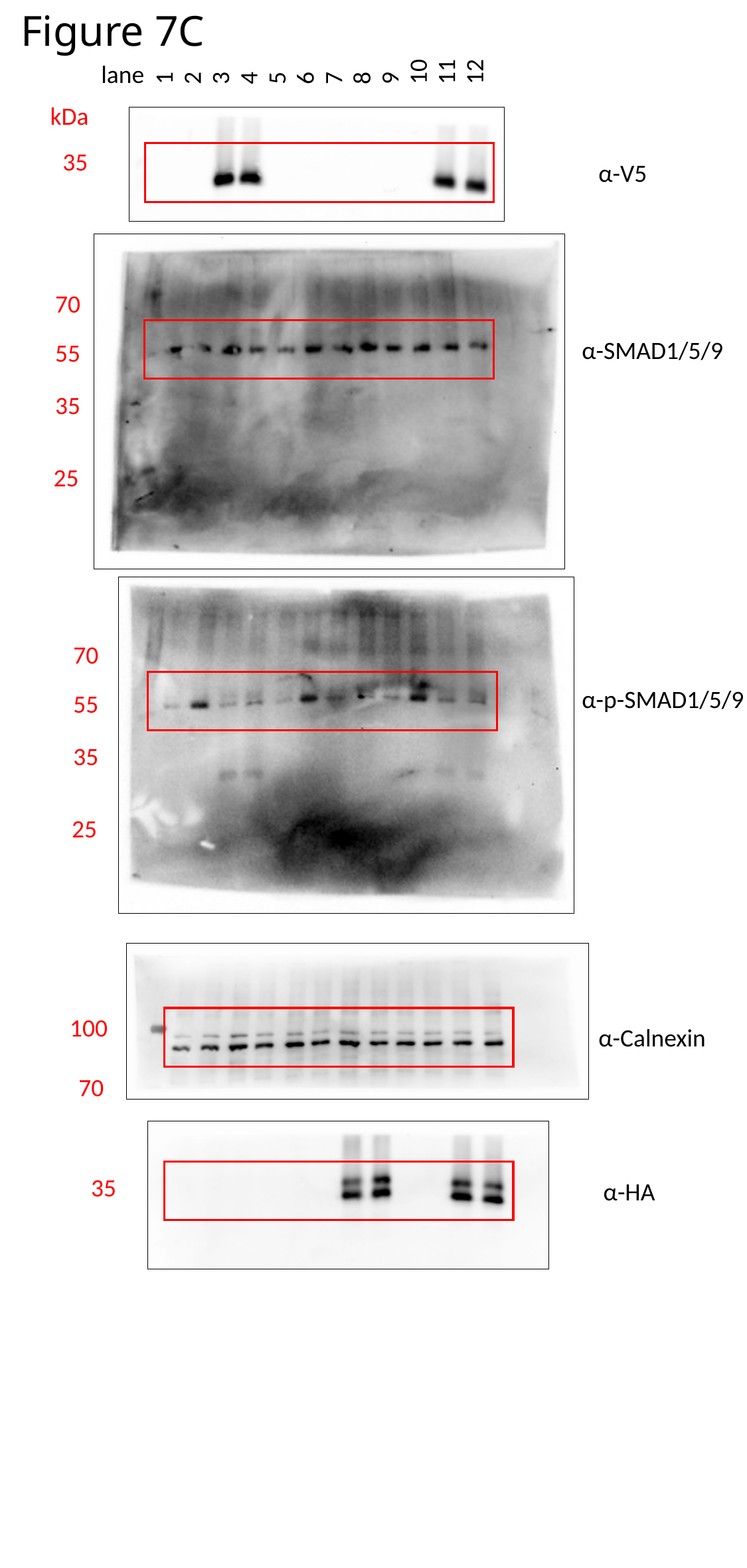

# Figure 7C
lane
1
2
3
4
5
6
7
8
9
10
11
12
kDa
35
α-V5
70
α-SMAD1/5/9
55
35
25
70
α-p-SMAD1/5/9
55
35
25
100
α-Calnexin
70
35
α-HA

Supplement: Supplementary file 6 — Source Data Fig. 7 [file 44319_2024_72_MOESM6_ESM.zip › 7C.pptx]

## Slide 1
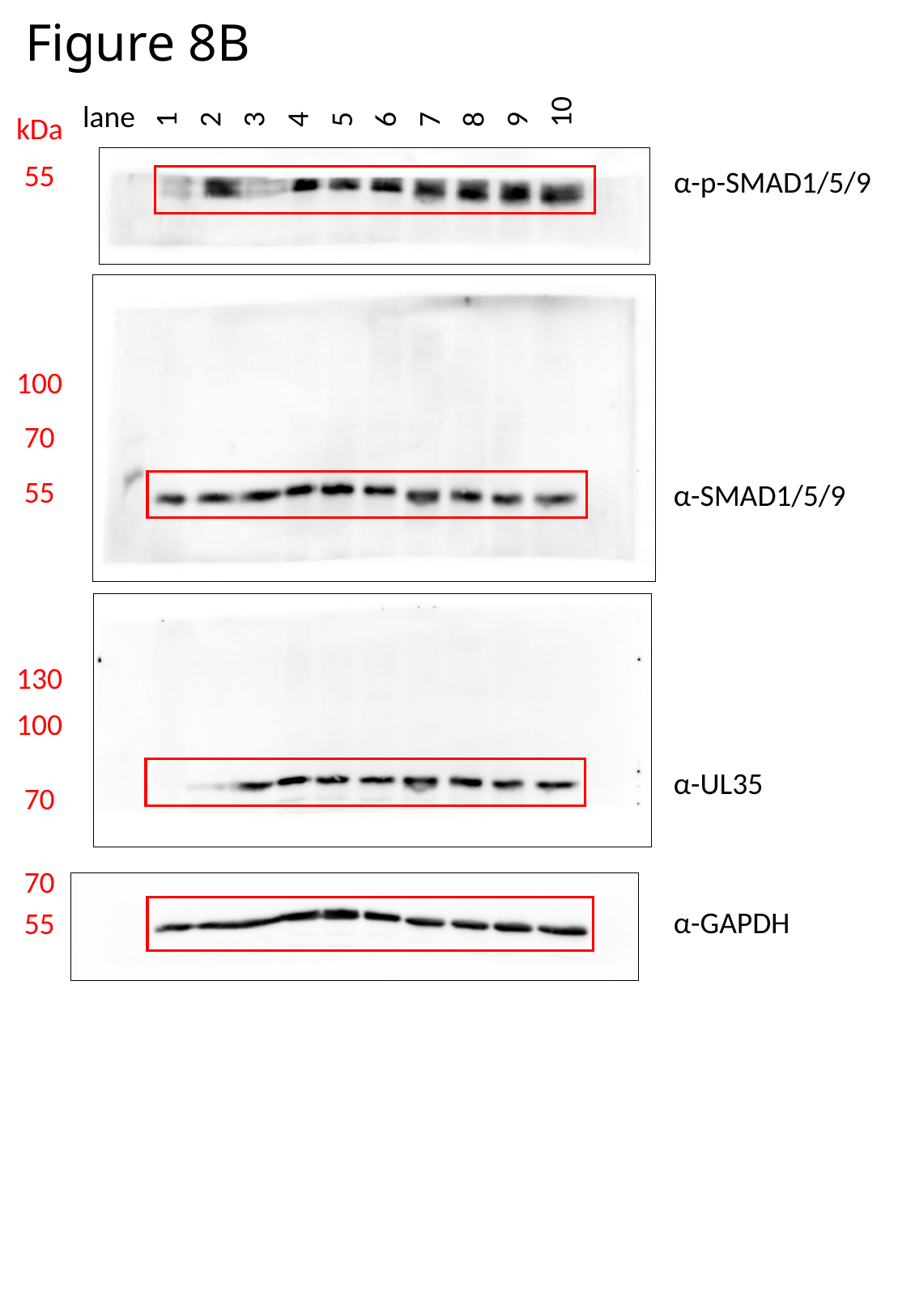

# Figure 8B
lane
1
2
3
4
5
6
7
8
9
10
kDa
55
α-p-SMAD1/5/9
100
70
55
α-SMAD1/5/9
130
100
α-UL35
70
70
α-GAPDH
55

Supplement: Supplementary file 7 — Source Data Fig. 8 [file 44319_2024_72_MOESM7_ESM.zip › 8B_image data.pptx]

## Slide 1
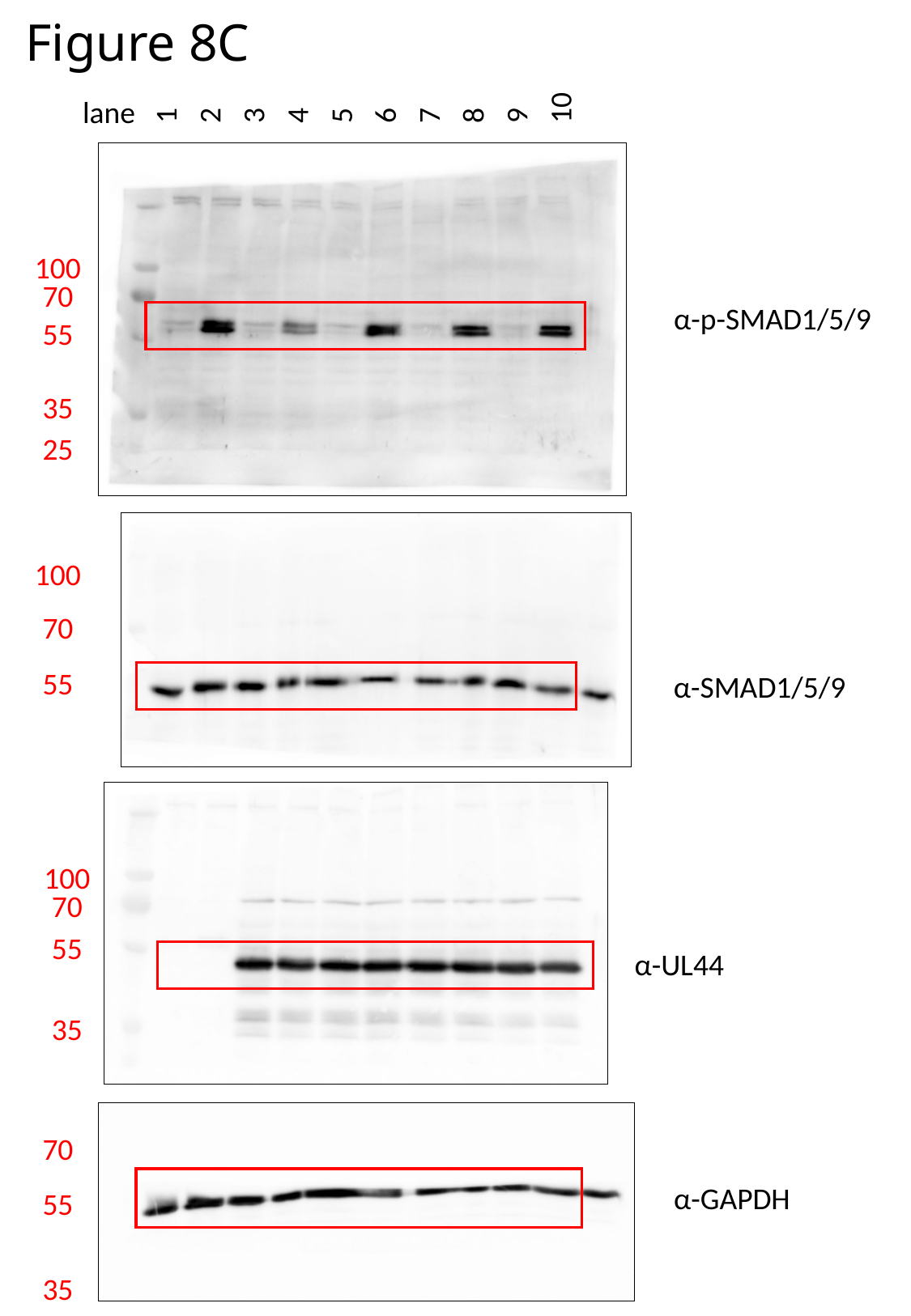

Figure 8C
lane
1
2
3
4
5
6
7
8
9
10
100
70
α-p-SMAD1/5/9
55
35
25
100
70
55
α-SMAD1/5/9
100
70
55
α-UL44
35
70
α-GAPDH
55
35

Supplement: Supplementary file 7 — Source Data Fig. 8 [file 44319_2024_72_MOESM7_ESM.zip › 8C_image data.pptx]
