# Supplementary material for: Novel role of bone morphogenetic protein 9 in innate host responses to HCMV infection
Source: EMBO Rep. 2024 Mar 11;25(3):1106–29. doi: 10.1038/s44319-024-00072-2 (PMC10933439; doi:10.1038/s44319-024-00072-2)
Supplement: Supplementary file 10 — Expanded View Figures [file 44319_2024_72_MOESM10_ESM.pdf]

Expanded View Figures

Figure EV1. Verification of BMP receptor expression and functionality of inhibitors.

(**A**) Presence of type I (ALK1, ALK2, ALK3) and type II (BMPR2) receptors in HFF-1 and 293T was verified by immunoblotting with the respective antibodies. Detection of GAPDH protein served as loading control. (**B**) Relative transcript levels of type I (ALK1, ALK2, ALK3, ALK6) and type II (BMPR2, ACVR2A, ACVR2B) BMP receptors in 293T were determined by RT-qPCR. (**C**) 293T were co-transfected with expression plasmids for the BRE-Luciferase reporter and a Renilla luciferase normalization control (EF1α-Renilla). 24 h post transfection, 293T were either stimulated with BMP9 (3 nM), or BMP9 (3 nM) incubated for 15 min at RT with an α-BMP9 antibody (1 μg/ml or 5 μg/ml) for 16 h, followed by a dual-luciferase assay readout. (**D**) 293T were co-transfected as in (**B**). 24 h post transfection, 293 T were either stimulated with BMP4 (18 nM), BMP6 (18 nM), BMP9 (3 nM), BMP15 (18 nM), or Activin B (4 nM), or with the ligands incubated for 15 min at RT with an α-BMP9 antibody (1 μg/ml) for 16 h, followed by a dual-luciferase assay readout. (**E**) HFF-1 were either mock, DMSO, Ruxolitinib (10 μM) or DMH1 (10 μM) treated for 2 h, followed by stimulation with IFNβ (5 ng/ml) or BMP4 (18 nM) for 2 h. Cells were lysed and lysates were subjected to immunoblot analysis with p-STAT1, STAT1, p-SMAD1/5/9, SMAD1, p-p38, p38, p-p44/42, p44/42, and Calnexin-specific antibodies. Data information: (**A–E**) Experiments were performed two independent times, one representative is shown. Luciferase fold induction was calculated by dividing Renilla-normalized values from stimulated samples by the corresponding values from unstimulated samples.

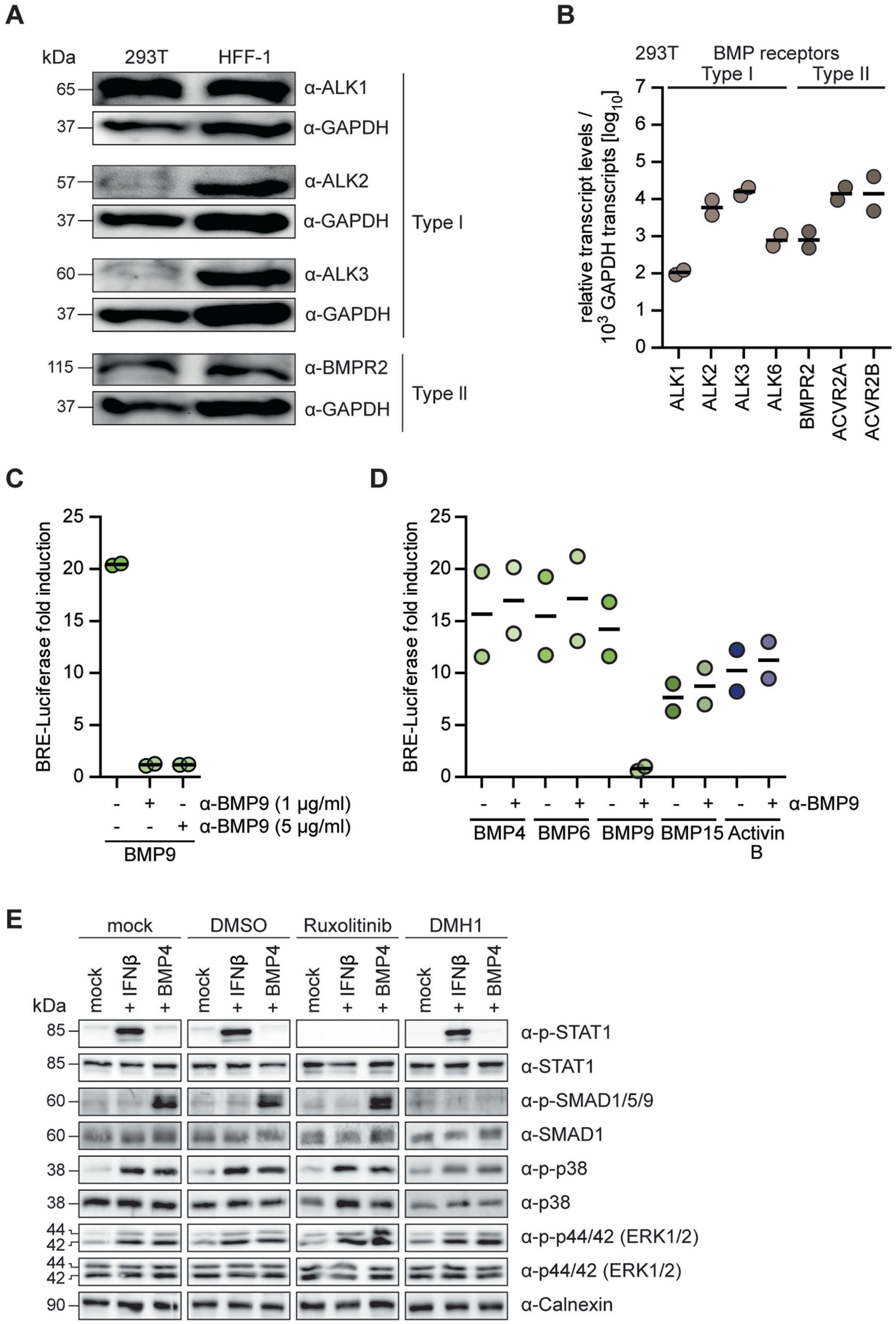
A
kDa
293T
HFF-1
65
α-ALK1
37
α-GAPDH
57
α-ALK2
37
α-GAPDH
Type I
60
α-ALK3
37
α-GAPDH
115
α-BMPR2
37
α-GAPDH
Type II
B
293T
BMP receptors
Type I
Type II
relative transcript levels / 10^3 GAPDH transcripts [log10]
ALK1
ALK2
ALK3
ALK6
BMPR2
ACVR2A
ACVR2B
C
BRE-Luciferase fold induction
α-BMP9 (1 µg/ml)
α-BMP9 (5 µg/ml)
BMP9
D
BRE-Luciferase fold induction
α-BMP9
BMP4
BMP6
BMP9
BMP15
Activin B
E
mock
DMSO
Ruxolitinib
DMH1
mock
+ IFNβ
+ BMP4
kDa
α-p-STAT1
α-STAT1
α-p-SMAD1/5/9
α-SMAD1
α-p-p38
α-p38
α-p-p44/42 (ERK1/2)
α-p44/42 (ERK1/2)
α-Calnexin

 © The Author(s)

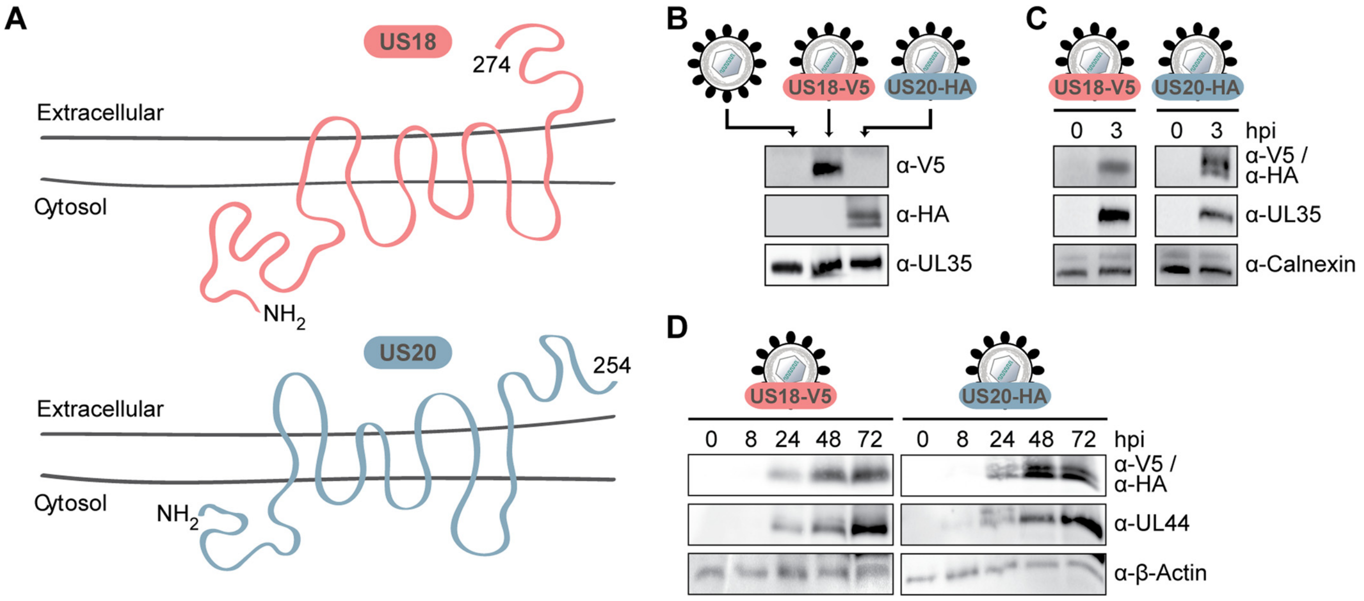

Figure EV2. HCMV US18 and US20 are associated with HCMV particles and are de novo expressed with early kinetics.

(**A**) Topology prediction of HCMV US18 and HCMV US20. NH_2 indicates the N-terminus, and the number indicates protein length in amino acids. Topology predictions for HCMV US18 and US20 were carried out using the online tools PredictProtein (https://predictprotein.org/), CCTOP (http://cctop.ttk.hu/) and DeepTMHMM (https://dtu.biolib.com/DeepTMHMM). (**B**) Recombinant HCMV expressing V5-tagged US18 or HA-tagged US20 were generated. 50.000 PFU of HCMV WT, HCMV US18-V5 and HCMV US20-HA were analyzed by immunoblot with antibodies for V5, HA and the HCMV tegument protein UL35. (**C**) HFF-1 were infected by centrifugal enhancement with HCMV US18-V5 or HCMV US20-HA (MOI 4). 3 h later, cells were lysed and cell lysates analyzed by immunoblotting with V5-, HA-, UL35- and Calnexin-specific antibodies. (**D**) HFF-1 were infected by centrifugal enhancement with HCMV US18-V5 or HCMV US20-HA (MOI 0.5) for the indicated time points. Cells were lysed and lysates analyzed by immunoblot with V5-, HA-, UL44- and β-Actin-specific antibodies. Data information: (**B–D**) Experiment was performed three independent times, one representative is shown.

© The Author(s)

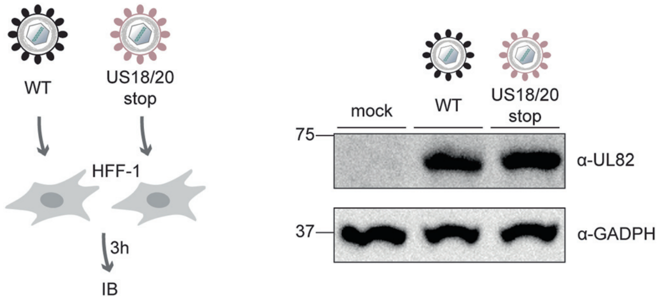

Figure EV3. The HCMV US18/20stop mutant infects HFF-1 with similar efficiency as HCMV WT.

HFF-1 were infected by centrifugal enhancement at MOI 4 with HCMV WT or HCMV US18/20stop and lysed 3 h later. Expression of the HCMV tegument protein UL82/pp71 was analyzed by immunoblotting with a UL82/pp71-specific antibody. GAPDH served as loading control. Data information: One representative of two independent experiments is shown.

© The Author(s)
